# 100 years of mathematical cosmology: Models, theories, and problems, Part A

**DOI:** 10.1098/rsta.2021.0191

**Published:** 2022-05-02

**Authors:** Spiros Cotsakis, A. P. Yefremov

**Affiliations:** ^1^ Institute of Gravitation and Cosmology, RUDN University, ul. Miklukho-Maklaya 6, Moscow 117198, Russia; ^2^ Research Laboratory of Geometry, Dynamical Systems and Cosmology, University of the Aegean, Karlovassi 83200, Samos, Greece

**Keywords:** universes, mathematical models, evolution of ideas, introductory survey

## Abstract

An elementary survey of mathematical cosmology is presented. We cover certain key ideas and developments in a qualitative way, from the time of the Einstein static universe in 1917 until today. We divide our presentation into four main periods, the first one containing important cosmologies discovered until 1960. The second period (1960–80) contains discussions of geometric extensions of the standard cosmology, singularities, chaotic behaviour and the initial input of particle physics ideas into cosmology. Our survey for the third period (1980–2000) continues with brief descriptions of the main ideas of inflation, the multiverse, quantum, Kaluza-Klein and string cosmologies, wormholes and baby universes, cosmological stability and modified gravity. The last period that ends today includes various more advanced topics such as M-theoretic cosmology, braneworlds, the landscape, topological issues, the measure problem, genericity, dynamical singularities and dark energy. We emphasize certain threads that run throughout the whole period of development of theoretical cosmology and underline their importance in the overall structure of the field. This is Part A of our survey covering the first two periods of development of the subject. The second part will include the third and fourth periods. We end this outline with an inclusion of the abstracts of all papers contributed to the Philosophical Transactions of the Royal Society A theme issue, ‘The Future of Mathematical Cosmology’.

This article is part of the theme issue ‘The future of mathematical cosmology, Volume 1’.

## 1. Introduction

Before we begin, we deem it fit to provide some comment on our choice of the somewhat restrictive word ‘mathematical’ in the title of this paper. We shall use the phrase ‘mathematical cosmology’ as almost indistinguishable to ‘theoretical cosmology’, ascribing to both an almost identical meaning. To us, the inclusion of material under both qualifications has an analogous interpretation and is closer in spirit to the two principles of selecting material for inclusion in their Course of Theoretical Physics used by Landau and Lifshitz (cf. [1], p. xi). We shall not deal with topics that may not be properly expounded without requiring at the same time a detailed account of the existing observational results, and secondly, we shall not discuss too complicated applications of the theory. (Both of these criteria are of course somewhat subjective to some extent.)

We shall then use the phrases ‘mathematical cosmology’ and ‘theoretical cosmology’ as having a very similar meaning, a possible difference being that of the amount of mathematical rigor contained in the presentation of the results. (We are well aware of the difficulties surrounding the general notion of ‘physical reality’ as it arises in physics, some of which are probably aggravated in cosmology. Traditionally, this notion is used to reflect differences between ‘theoretical’ and ‘mathematical’ physics, but we choose not to enter into such a discussion in this paper.) The emphasis in this approach is perhaps almost orthogonal to the more popular approach followed nowadays, the data-driven, empirical or ‘actual cosmology’ (for the latter, see [2] for a standard treatment).

In the following sections, we shall present a panorama of cosmological models that aim at describing some aspect of the universe. But what is a cosmological model? The standard definition of a cosmological model in relativistic cosmology (see, e.g. [3] and refs therein), although extremely helpful for giving orientation and meaning to a very wide set of issues in cosmology, is perhaps too narrow for our present purposes.

Such definitions (or similar ones given in [4,5]) present cosmological models as having a timelike (fundamental) vector field together with a manifold and a spacetime metric which satisfies the Einstein equations. Apart from the fact that general relativity is currently only one out of many different geometric ways of describing an interaction of spacetime geometry and matter, there are areas in cosmology where even the very notion of spacetime and that of a fundamental timelike field are probably too restrictive to use. As typical examples of this, we may mention the multiverse, the landscape or the notion of braneworld, all of which require other types of geometry and dynamics for their description rather than that found in the standard definition of cosmological model.

The beauty and enormous variety of ideas of modern mathematical cosmology have their roots in the different kinds of geometry required to be developed and studied in parallel and in conjunction with those cosmological ideas for a better understanding of different aspects of this most majestic of fields of theoretical physics.

The purpose of this paper is twofold. Firstly, we describe some important key developments in the field of theoretical cosmology since its modern beginning in 1917. We single out and discuss some of the important ideas that characterize the nature of this field within theoretical physics. Secondly, we provide a short description of the contents of the theme issue ‘The Future of Mathematical Cosmology’, by providing the titles and abstracts of the particular contributions.

The plan of this paper is as follows. We split the whole period of development from 1917 until today into Parts A and B, and this part will cover the first two periods up to around 1960. Part B will include the remaining time and will be published separately. In the total of 36 subsections contained in both parts of this survey, we give brief descriptions of the emergence, development, importance and interconnections of most major subfields of theoretical mathematical cosmology, such as relativistic, modified gravity and dark energy, inflationary, quantum, string, M-theoretic and brane cosmologies; we also discuss alternative domains and threads of development such as singularities, horizons, measures, stability, genericity and topology. We focus on key theoretical discoveries as well as fundamental ideas that were to become (or, in fact, may become) instrumental in the development of the whole field. This is presented in four key time periods ranging from 1917 to today. In this perspective, at the end of each part of this paper, we comment on the contents of individual invited contributions to the theme issue ‘*The Future of Mathematical Cosmology*’ that are included in two separate volumes of the Philosophical Transactions of the Royal Society A.

## 2. First period, 1917–1960

In the first period of developments of mathematical cosmology, there appeared a great number of novel fundamental ideas that still play a major role today in shaping the field of theoretical cosmology:

— The ‘universes’ as solutions of the field equations
— The cosmological constant and vacuum energy
— Homogeneity of the universe
— Inhomogeneous and anisotropic cosmologies
— Expansion and contraction
— Evolution vs. steady state
— Big bang vs. bouncing models
— Gravitational stability and perturbations
— Hot big bang
— Causality and time travel
— Local vs. global structure

All these ideas are as fundamental and important in theoretical cosmology research today as they were in the beginnings of modern cosmology. It is an amazing fact that the subsequent development of the field in the coming decades proved that all these novel and important ideas are still playing a major role, but at the same time are a small part of the complex network of methods and directions that constitute mathematical cosmology today.

### (a) Einstein static universe

On 8 February 1917, Einstein announced the first application of general relativity to the universe considered as a whole, the *Einstein static universe*^1^ [8]. It was a cosmological model of motionless matter, having positively curved spatial sections (so had a finite volume but no boundary) and an added repulsive force (dependent on the cosmological constant) balancing the attractive gravitational force, resulting in a static world. But perhaps the most distinctive new feature of Einstein’s static universe was its assumed overall homogeneity. In other words, in Einstein’s proposal, there is an upper bound—provided by large-scale homogeneity—to the hierarchical structure in all of physics starting from elementary particles, atoms, molecules, planets, etc, and ending with large-scale uniformity. (For the opposite view, that of *no* upper bound to the hierarchy of clusters of galaxies, see §c.)

Einstein’s static world remains always static because of the presence of Einstein’s cosmological constant—the λ term—a new, additional term in the original Einstein’s equations of general relativity that he introduced for that purpose. The spherical nature of Einstein’s static universe implies a maximum (spherical) distance around the universe, the so-called circumnavigation time, equal to $t_{ct}=2\pi a$, a being the scale factor (or, radius of curvature). Then from the field equations, it follows that $t_{ct}∼\sqrt{4/\rho }$ hours (in suitable units), so that this time is increasing with decreasing density, being equal to about 2 h for the water density. The propagation of light in an Einstein static universe (as seen in the usual spacetime diagram depiction of it, the ‘Einstein cylinder’), implies that this model acts like a lens, antipodal points are seen nearby to each other by observers situated in them, and receding images from a point in this universe first appear smaller until they reach its antipode, when they look bigger.

But perhaps the most important property of Einstein static universe is its instability against homogeneous and isotropic perturbations discovered as well as other types of fluctuations a few years later (see below). This important instability property reveals that Einstein’s world can only be one of a *transient nature*, and depending on its matter content, it can connect with other eras of the cosmological history (see §4).

Einstein’s idea was truly extraordinary. By extension, every solution of the field equations of general relativity would describe some possible universe, and here was the first one. This discovery marked the beginning of cosmology: the ‘universe’—in this case Einstein’s static solution—comes equipped with a variety of definite ‘physical’ properties as predicted by general relativity, it is homogeneous (and isotropic), finite, spatial closed (with topology that of the 3-sphere), positively curved, having a nonzero matter density, and static.

This led Einstein to struggle with the dilemma: there was apparently only one Universe, but an infinite number of *universes*, that is unknown *solutions* to the mathematical equations of general relativity describing possible models of the Universe; so how could one reconcile the two? Also how was it possible to control the universes’ behaviour at infinity, if that was allowed elsewhere, or avoid having a finite edge? A vast field of investigation had just opened.

### (b) De Sitter spacetime

Very soon the second universe, solution of the modified Einstein equations with the lambda term, was found by W. de Sitter [9]. That described an empty (zero matter density) space, static model as was originally proposed by de Sitter. It was later realized that it may also be described as a model for an expanding universe in both time directions, because of the negligible effect of gravitational attraction. In fact, it is constantly accelerating with time due to Einstein’s cosmological constant term introduced earlier the same year that de Sitter spacetime first appeared.

The *de Sitter universe* has no beginning or end. This universe plays the role of an ‘undisturbed’ state for the modified Einstein equations with a cosmological term, in a similar way as does Minkowski space for the original field equations (i.e. with λ=0). However, de Sitter space is really different to Minkowski space in certain quantum aspects, see below. It also plays a fundamental role as possible asymptotic states of more general homogeneous cosmologies (see later sections below).

In fact, there are two ‘de Sitter’ spaces, one for λ positive—*the* de Sitter space (dS)—and the case of negative λ, the so-called anti-de Sitter space (AdS) (see [10] for an excellent discussion of these spaces, and [11] for a more specialized description of their infinity properties).

Perhaps the simplest way to distinguish dS from AdS universes is that dS, like Schwarszchild spacetime but unlike AdS, has a horizon separating its static regions from its evolutionary ones. This was not clear initially, but it later became understood with the work of Eddington and Lemaître. However, unlike dS space, the AdS spacetime contains closed timelike curves and is globally static. Normally, when we talk about AdS we simply mean its universal cover, a space without closed timelike curves. dS and AdS spaces are very important in current primordial cosmology, especially in braneworlds and holographic models of the early universe (see section on branes in Part B of this survey). dS space also has an instability, classically forbidden, associated with the possibility of defining a non-thermal temperature using the event horizon [12]. In this case, one may use the Schwarzschild-de Sitter (Kottler) solution that describes a black hole immersed in de Sitter space, to construct Euclidean instanton solutions with special properties (cf. [13] and refs. therein).

### (c) Fractal universes

Einstein’s static universe was the first application of general relativity to cosmology, and, in fact, it created such a boost to the study of universes that an application of the old Newton’s theory to cosmology seemed an impossibility. However, in 1922 the claim of Einstein of an upper bound to the galaxy hierarchy was to be challenged by C. Charlier [14], who introduced a *fractal universe* as it may now be called—a distribution of a never-ending repetition of clusters *ad infinitum*, on larger and larger scales (the word fractal was introduced much later [15]).

Charlier’s universe possessing a fractal hierarchy was based on earlier ideas of F. d’Albe and others (see [16] for an early history of such ideas) and was arranged such that, although the overall density and volume were infinite, the *average* matter density was zero, thus bypassing the obstacles of the ‘gravitational paradox’. (According to the Newton-Ivory theorem [17], at infinite distance in an infinite universe of constant density, the Newtonian force may take arbitrary values.)

Also, it has the same clustering on all scales, a property avoiding the difficulties associated with Olbers’ paradox, that is the integrated luminosity of all stars at every place in the universe is now finite—the night sky is still dark. In addition, the gravity pull is everywhere finite and so star velocities remain small at all scales.

The Charlier model of an inhomogeneous, infinitely large, fractal universe with infinite mass but zero average density was soon after treated in a more elaborate way by F. Selety [18], and more recently by others [19,20], but an objection (originally raised by Einstein) was how such a hierarchy could arise in the first place, remained unresolved.

More recently, attempts to explain the large-scale clustering by a fractal universe have re-emerged [21–23], although conflict of such explanations with the large-scale observed homogeneity from the temperature fluctuations in the cosmic microwave background (hereafter CMB) is taken as an indication that clustering cannot continue indefinitely.

### (d) Friedmann solutions

The true gallery of possibilities allowed by the general theory of relativity was first discovered by A. Friedmann in 1922–24 [24,25]. Friedmann discovered a family of universes that were homogeneous and isotropic, having matter, non-zero curvature, as well as non-zero cosmological constant. He found out that they are described by equations containing the previously found solutions as well as many other novel additional universes. (Today Friedmann universes are also called Friedman–Robertson–Walker, Robertson–Walker, Friedmann–Lemaître, or even Friedman–Robertson–Walker–Lemaître solutions. We shall use the notation ‘FRWL’ below.)

The non-static character of the *Friedmann universes* fitted well with 1912 observations of V. Slipher [26] showing a red shift in the wavelengths of distant nebulae (later coined galaxies), as did de Sitter’s universe, but no one at the time was ready to accept these results as showing an overall expansion of the universe. These universes are models with a perfect fluid source and may or may not have a cosmological constant (the name Friedmann–Lemaître is sometimes reserved for the latter case).

### (e) Lemaître universe

The connection of the dynamical character of Friedmann models with the observations showing an overall expansion was done a few years later. G. Lemaître in 1927 [27] combined the Friedmann models with the phenomenon of redshift, calculating for the first time the constant in the ‘Hubble’s law’ v=Hr, connecting the distance to recession velocity, found by Hubble two years later.

The 1927 paper of Lemaître was translated into English in [28] after a suggestion of Eddington (for this amazing story that made Lemaître ‘the most well-known theoretical cosmology of the day’, see [29]). In that same 1927 paper, Lemaître had also shown that the Einstein static universe was unstable, small irregularities would in fact grow in time. The resulting *Lemaître universe* has the property of coasting as static an infinite time in the past, before expanding and accelerating like de Sitter’s universe in the future (this instability of the Einstein static world was also shown independently a few years later by A. Eddington [30], and this universe is sometimes called the Eddington–Lemaître cosmology).

Sometimes by Lemaître universe, we mean one that starts with a big bang (reserving the infinite coasting static period for the Eddington–Lemaître model), then passes through a finite hesitation period where it is described by the Einstein static universe and then ends with a ‘whimper’ expanding like a de Sitter space into the future.

Lemaître also appears to be the first to connect the cosmological constant to the energy of the vacuum in a paper published in 1934 [31], now related to what is termed dark energy.

### (f) Einstein-de Sitter universe

But the situation with a zero cosmological constant was no less interesting. Einstein and de Sitter jointly in 1932 emphasized the importance of what is perhaps the simplest of all the Friedmann solutions, the *Einstein-de Sitter universe* as it is now called [32]. It is a flat model starting at a finite time in the past, and expanding at a critical rate with scale factor proportional to $t^{2/3}$ and having zero cosmological constant.

Today, this model agrees with observations so greatly that it has become a problem to explain why this is so and why instabilities have not ruined it one way or another (‘flatness problem’).

### (g) Tolman’s bouncing universe

Another distinctive aspect of some of the Friedmann solutions with zero lambda is their symmetric or *cyclic character*, as they evolve from small to large to small, and a natural question suggested itself: the possibility of an infinite number of such cycles.

This was taken seriously in *Tolman’s bouncing universe* of 1932 [33], who discovered that such a possibility, in the case of zero cosmological constant, must be accompanied by an increase in the overall entropy of the universe from cycle to cycle, increasing the maximum of the expansion in a cycle relative to the previous one. This is in sharp contrast to the idea of a beginning in a finite time in the past and end in the future.

Oscillating universes like Tolman’s play an important role in theoretical cosmology today. However, the main issues that should be addressed in such models is the ever-increasing temperature of the CMB from cycle to cycle, as well as the amount of radiation and the entropy. We shall see later that the Tolman’s model may be useful to motivate the existence of wormholes.

This concludes the various properties of the Friedmann universes which, although being the simplest class of cosmological model, is one of the most important and must be thoroughly understood in any cosmological theory before embarking on the study of more complicated models.

### (h) Kasner models

A novel feature of possible universes with no cosmological constant was discovered by E. Kasner as early as 1921 [34], an expanding universe starting a finite time ago but having the property of *evolving in time at different rates in the three different spatial directions*. Kasner’s empty and flat universe may contract in one direction while expanding in the other two, keeping a constant volume. It is *anisotropic*. In the past direction, neither matter not a lambda can alter its behaviour and avoid a singularity, but in the future, a matter-filled Kasner model looks like Einstein-de Sitter, while a ‘lambda-Kasner’ universe looks like de Sitter.

In addition, the Kasner universe plays a significant role in many parts of cosmology. Its late-time asymptotics (as time approaches infinity) are isotropic, as they approach the Einstein-de Sitter, and so Kasner may be a suitable model for the real universe. At early times however, it contracts to the singularity anisotropically: it can do so either with two of the orthogonal directions contracting and one expanding, resulting in a *cigar* singularity. On the other hand, if two directions are expanding and one contracting, the model develops a *pancake* singularity. These kinds of singularity are absent in the simpler FRWL models. We also note that in this special case, the expanding direction has the property of allowing communication around it in a cylinder, the model develops no horizons along that direction.

The Kasner universe is the simplest cosmology of a much wider family of cosmological models, the Bianchi universes, which we discuss below. It is very important in current studies of the cosmological singularity because it appears that some form of ‘Kasner behaviour’ must be taken into account in describing the singularity.

### (i) Lemaître-Tolman universe

Then, a joint development really took things to the next level. A common feature of all universes discussed so far (except the fractal hierarchical universe) is their large-scale homogeneity, either in the isotropic FRWL universes or in the anisotropic Kasner spacetime. But, the first discoveries of *inhomogeneous universes* soon came along. The *Lemaître-Tolman universe* [35,36] contained under- and over-densed regions rather than some average density, with the rate of expansion, curvature and matter density depending on the spatial points, making the overall evolution of the universe more and more inhomogeneous with time.

These universes would locally collapse to form galaxies (or ‘⋯dust particles (nebulae) which exert negligible pressure⋯’ as Tolman calls them in [36]), and being globally inhomogeneous would look totally different than our local homogeneous patch.

A similar inhomogeneous universe was found by Einstein and E. Strauss in 1945 [37], the so-called *Swiss-cheese universe*, a spherical, pressureless model in which spherical regions were removed and equal masses were put back at the centres of the removed regions. Because of spherical symmetry, there are no gravitational waves in this model, but this feature was later rescued by replacing with cylindrical symmetry, as in the later Einstein–Rosen solution [38].

More generally, the importance of inhomogeneous solutions in cosmology cannot be overestimated. One sees that even the earliest known solutions like the Swiss-cheese model introduce a level of sophistication higher than the simplest homogeneous solutions, in that they introduce new problems not met before, but also the problem of how to join the solutions at their common boundary, *the junction conditions.* This in turn plays a key role in many areas of modern cosmology, such as the deformation geometry and evolution of strings, branes, and higher-dimensional membranes propagating in spacetime (cf. e.g. [39,40], and refs, therein), not to mention quantum cosmological aspects (cf. e.g. [41]).

### (j) Lifshitz universes

The exact solutions considered in the previous subsections were useful in turn because they pointed to the general problem of how to combine anisotropies as in the Kasner world, and inhomogeneities as those present in the models of Einstein–Rosen–Strauss.

Mathematically, this is related to the problem of how to carefully study all possible *small perturbations* of the homogeneous and isotropic universes of Friedmann. This was first done in *E. Lifshitz’s theory* [42] of first-order cosmological perturbations, a landmark study in 1946. Lifshitz’s analysis revealed that the Friedmann solutions have the hidden property of *gravitational instability*; they share atypical, nongeneric features, not present in a more general situation. A further analysis that appeared later treated not only fluctuations inside the horizon (that is smaller than the radius of curvature) but also those comparable to it, cf. [43,44], Sect. 115. For the second-order theory and recent work, see [45,46], and for an advanced textbook treatment [47], chap. 5.

This study also set the stage for all future research on the problem of the origin and development of small cosmological fluctuations, arguably perhaps the most central problem of contemporary cosmology. This is so because through the field equations, it is the basis of the issue of the formation of structure in the universe, namely, how irregularities that originate in the early universe will develop coherently starting from small initial disturbances. This is a very involved issue connected with other major and not completely settled problems such as the horizon and flatness problems (for a thoughtful presentation, see, as for many other topics in cosmology, the excellent ‘introductory’ treatment in the book [16]).

### (k) The hot big bang

But at the time of the inhomogeneous studies of Lifshitz, even in the homogeneous picture the last word had not been said yet. It was not until 1948 that G. Gamow [48] and two of his students, R. Alpher and R. Herman, proposed that *the early universe was not only very dense but also very hot* and showed that in such a universe the ratio of the matter density to the cube of the temperature of heat radiation is a constant (see [49,50] for an insightful presentation of their findings).

Hence, if one measured that density and ratio today, they could find the present temperature of the leftover radiation. The authors gave an estimate of this temperature about 5 degrees Kelvin, but no one gave any serious attention to this result back then and went completely unnoticed, despite the fact that this prediction was no other than that of the later discovered cosmic microwave background thermal radiation left from a very early stage, the *hot big bang*, in the evolution of the homogeneous universe.

This is a tremendous result equivalent to the discovery of a radiation era in the early universe with density exceeding that of matter. Also, this radiation survives until today with a much cooler temperature. It took more than 15 years until the cosmic microwave background radiation, observationally discovered by Penzias and Wilson in 1965, convincingly explained by R. Dicke and his group the same year, and accurately measured to be isotropic as late as 1967.

This discovery was perhaps the single most important one in theoretical cosmology as it established it as a physical science (see [2], chap. 4, for a very detailed analysis of the various developments in the long period leading to this discovery). It also led, with the help of particle physics, to *the standard model of cosmology*, where a detailed history of events during the various periods in the history of the expanding universe may be built. This history extends from the period of the early universe (sometimes this period is also called ‘the big bang’) for about 55 orders of magnitudes from the Planck time until the beginning of the current matter dominated era which lasted only for about 5.5 orders of magnitude (steps measured in logarithmic time). For a popular account of the standard model of cosmology, we refer to [51], and to [5] for a detailed presentation.

### (l) Steady-state universes

However, not all ideas during the first period in the development of theoretical cosmology were about the big bang. The steady-state universe of *continuous creation* by H. Bondi, F. Hoyle, and T. Gold in 1948 [52,53] introduced the so-called perfect cosmological principle, a variant of the homogeneity principle that Einstein had introduced earlier in his static model, in which the universe looks the same not only in space but also for all times.

That was a completely different model from any big bang theory, eventually based on a different geometric principle we shall later analyse, the scalar-tensor theory (see [54] and refs. therein, for a discussion of steady-state models from that angle).

Not being static but ‘steady’ (or stationary), that is eternally expanding with constant acceleration, the state of the universe required matter to be continuously created in time, instead of it being created in a unique time as in the big bang model (incidentally the name ‘big bang’ was coined by Hoyle during 1949 in a BBC radio program). Such ‘creation theories’ have matter to be created not from radiation but apparently out of nothing. This is to be compared with modern attempts to describe spontaneous creation ‘out of nothing’.

Steady-state cosmologies sharing the feature of continuous creation include the one by W. McCrea proposed in 1951 [55], see also [56], that incorporated this idea within general relativity as cosmic tension (negative pressure, p=−ρ) leading to a global state of constant density. This ingenious argument of McCrea implied that expansion continuously creates new matter to keep a constant mass density. (This is again to be compared with the inflationary idea of expansion at constant density coming from a false-vacuum.)

These ideas were considered as rivals to the Friedmann models for a period, until it was found in 1952 by W. Baade that previous astronomical calculations of distance calibration were in fact wrong by a factor of two or more, a fact that erased the initial motivation for such alternative models. For a modern reincarnation of the steady-state idea, see [57].

### (m) Gödel’s universe

Another important advance in theoretical cosmology before the 1960s was *K. Gödel’s rotating universe* published in 1949 [58], a zero-pressure fluid solution to Einstein’s equations with a negative cosmological constant. This solution for the first time allowed time travel into the past in exchange with relativistic speeds and exotic matter configurations.

A failure of a cosmic time function existing for all fundamental observers (able to synchronize their clocks) implies that t=const. Hypersurfaces in spacetime cannot be spacelike everywhere and will therefore turn timelike somewhere. This leads to causality violations, such as closed timelike curves crossing such hypersurfaces an odd number of times, and in Gödel’s model this situation is generally provided by a non-vanishing vorticity in the timelike direction, cf. [11,59,60] for more details.

More recently, however, some authors have extended the original solution to *causal Gödel universes*. In these cosmologies, closed timelike curves are absent in Gödel-type models in geometric extensions of general relativity such as when a massless scalar field is added [61], in a higher-order gravity theory quadratic to the Ricci curvature [62], and in various string theories [63], or even hidden behind a holographic screen [64].

Gödel’s universe was instrumental in the further development of modern theoretical cosmology because, despite its incompatibility with the observations about the expansion of the universe, clearly provided the first example of the basic distinction between local and global properties in cosmology.

### (n) The many universes

An efficient way to classify cosmological models is via the consideration of an isometry group acting on spacetime, thus defining an orbit through each spacetime point, i.e. submanifolds in spacetime where all physical and geometric properties remain invariant. Isometry, or symmetry, groups signify how symmetric a given cosmology is.

Roughly speaking, the dimension r, of an symmetry group acting on a spacetime of dimension n, splits into a homogeneous part—the orbit of dimension d—and an isotropic part of dimension s, such that r=d+s. In this situation, d signifies the degree of inhomogeneity and s that of isotropy.

For example, in a four-dimensional spacetime (n=4), d=4 implies an unchanging world in space *and* time, d=3 a spatially homogeneous universe, and for d≤2, that is d=2,1,0, we have inhomogeneous universes, the last case corresponding to the real universe—no symmetry. For s=3, we have complete isotropy, s=1 is the so-called locally rotationally symmetric (LRS) universes, while s=0 is the case of no isotropy (the case s=2 is not possible). For a textbook treatment of the classification of universes in terms of a symmetry group (see for example, [3], Part 4, and refs. therein).

A. Taub [65] studied the Einstein equations using group theoretic ideas for the first time in 1951. He exploited a classification of spacetimes into nine families discovered by L. Bianchi in 1897, the so-called *Bianchi universes* [66] (this is the case s=0,d=3). He discovered that all previously known homogeneous FRWL solutions of the field equations could be easily accommodated in that classification, but there were many more solutions, like those discussed above, whose cosmological study was to occupy the coming decades.

One new feature of the Bianchi universes was the possibility of gravitational waves, as well as new types of early- and late-time evolution. These universes are all homogeneous but anisotropic; the Kasner model is described by the simplest one of them, the Bianchi type I, and so could accommodate other features not present in simple isotropic ones, like shear, vorticity, and anisotropic curvature, with respect to an overall isotropic Hubble flow.

The Bianchi cosmologies also play a role as asymptotic states of more general and more realistic inhomogeneous spacetimes. One particularly interesting class of exact inhomogeneous cosmologies are the Szekeres–Szafron models which may be regarded as nonlinear perturbations of the FRWL universes.

The real difficulty in dealing with inhomogeneous models is their different geometry. For an introduction to inhomogeneous cosmology, see [3], chap. 19, and for a full discussion of the known inhomogeneous models, see [67]. In these situations, one also needs to have a well-defined, useful and general formalism for cosmological models. This issue has long been an important one in mathematical cosmology, as it is not a good practice to invent a new formalism every time one studies a different cosmological model. For an introductory discussion of the most well-developed formalism for general cosmology, see [3], Part 2, and also [68] and refs. therein.

## 3. Second period, 1960–1980

Novel ideas that emerged in this period with special relation to cosmology can be collectively summarized as follows:

— Geometric extensions of general relativity
— Singularity theorems, global techniques
— BKL conjecture
— Mixmaster universe and horizons
— Particle physics and classical singularities

### (a) Brans-Dicke theory

Building on earlier and more speculative ideas of P. Dirac on the variation of Newton’s gravitational constant G [69], Brans and R. Dicke [70] in 1961 came up with an ingenious theory of gravitation and a family of universes that proved cosmology was possible outside the realm of general relativity. The *Brans-Dicke gravity theory* predicted universes which were expanding and contracting pretty much as in Einstein’s theory, but their properties depended on the values of gravitational constant G and its time-dependence through a new scalar field in addition to the metric, the primary function of which is the determination of the local value of G.

This scalar field is of purely cosmological origin, as it is not one of the curvature invariants formed by the curvature tensor which fall off more rapidly than $r^{-1}$ from a mass source, and so become unsuitable for cosmology as they are determined by nearly rather than distant matter.

This is a distinctive feature of this theory, the mediation of gravitation at cosmological distances by a scalar ‘degree of freedom’ in addition to the tensorial degrees of freedom, resulting in three degrees of freedom rather than two in general relativity. So, one does not expect to find this scalar field in solar system tests, because it is supposed to be of cosmological origin (in the same way that one does not apply solar system metrics to cosmology), cf. [71]. However, of course, solar system tests constrain the values of the BD parameter ω to values bounded below by 6 (cf. [69] equation (36)), or higher in more recent studies. It is also important that in BD theory ω be positive and of order one if contributions of nearby matter to the inertial reaction are to be positive [69].

As a rule, any solution of general relativity with matter having energy-momentum tensor with a vanishing trace is a solution of the Brans–Dicke theory. In addition, flat perfect fluid solutions approach the vacuum solutions at early times, whereas the late-time behaviour is ‘Machian’ in the sense that matter dominates over the kinetic energy of the scalar field [72].

For anisotropic extensions of BD cosmology including the possible singularity removal, see [73], for an early description of the possible (non-)occurrence of oscillatory behaviour in BD models where a vector field in addition to the BD scalar is included; see [74,75], and for more recent qualitative approach to dynamical stability in the space of all (isotropic) solutions, see [76]. For interesting reviews of the early history of BD theory with much wider commentary and ideas on the influence and importance of scalar fields in cosmology, see [77,78].

This was the first example of a cosmological theory qualitatively distinct from general relativistic cosmology, still perfectly reliable. The Brans–Dicke gravitational field is *mediated* by a scalar field in addition to the spacetime metric, and Brans–Dicke theory has the property of being equivalent to general relativity under a conformal transformation of the fields [79]. In this ‘Einstein frame’, distinct from the original frame of the Brans–Dicke equations—the so-called Jordan frame’—the theory appears in the conventional form with the scalar field playing the role of a matter field. Then, the remaining masses of all particles are affected by their interaction with the scalar field, which thus reduces their masses. This led to a novel interpretation of the smallness of G: this is interpreted as small because the masses are conformally reduced drastically by their interaction with the field which is generated by all the matter at cosmological distances [79].

In any of these two interpretations, the scalar field gives a time-dependent Newton’s constant G, and so Brans–Dicke theory appears as a framework for the construction of concrete models for the time-variation of the ‘constant’ G during the evolution of the universe. For a discussion of the new and intriguing possibilities of varying constants in theoretical cosmology, see [80,81].

Starting in the 1980s, and perhaps with the input of string cosmology, other cosmological extensions of general relativity began to attract serious attention. The whole family of possible gravitational actions prevailed under the name *scalar-tensor cosmology*. Based on early work of P. G. Begman, R. V. Wagoner, and K Nordtvedt on extensions of the Brans–Dicke prototype theory, various possibilities appear with their myriad of possible couplings between matter fields, scalar fields, and gravity (see, e.g. [82,83] for the ‘relaxation’ problem of such theories to general relativity, with refs. to earlier work).

A basic characteristic of scalar-tensor theories is that the original BD dimensionless coupling constant ω is promoted to a dimensionless coupling *function* of the scalar field, thus hugely enriching the dynamical possibilities (and problems!) of the original BD theory. Scalar-tensor theory, in particular ‘pure’ BD theory also appears to be related to string theory, the latter predicting, however, a non-minimal and non-universal coupling to the BD scalar (coined the ‘dilaton’ in string contexts), cf. [84].

### (b) Torsion and cosmology

A different type of geometric alternative to general relativity with additional fields that appeared very early in the 1960s was the *Einstein-Cartan gravity theory*, a mixture of the structures of general relativity with another theory introduced by Elie Cartan [85] in 1924, and based on two invariants of the Poincare group—the space-time curvature and *torsion*. In solid-state physics torsion was used to describe dislocations in crystals, however, attempts to include torsion in Einstein’s already well-studied and developed theory of gravity appeared only in the 1960s. The first people who recalled torsion were, apparently, Kibble [86] and Sciama [87], in whose works an original idea was voiced about the possible relationship of space-time torsion with the proper angular momentum of matter.

But the theory began to develop only after the first (and sensational) torsion effect was calculated. In the works of researchers of the Krakow school Kopczynski [88] and Trautman [89], it was demonstrated that the torsion of space-time, which geometrically reflects the fact of polarization of spin of the dust particles, the source of the gravitational field, can eliminate cosmological singularities in Friedmann universes. The Einstein–Cartan theory has two essential features: (i) the torsion field equations (obtained by varying the Lagrangian by affine connection) are purely algebraic equalities linking torsion with its source, the spin of particles of gravitating matter; this means that torsion does not extend in this theory. (ii) Since the same Lagrangian (scalar curvature) with a single coupling constant (the Newton–Einstein gravitational constant) is used to obtain the equations of the gravitational field and torsion, the spin-torsion interaction turns out to be proportional to this constant and is therefore extremely weak. In addition, the weakness of this interaction is repeatedly ‘aggravated’ by the Planck constant, which in the classical version of the theory is linked with the spin value of the gravity source. Also much smaller are the effects of ‘torsion repulsion’, proportional to the square of the torsion components.

In the Einstein–Cartan theory, generalized in spaces with affine connection, the components of the torsion tensor in the simplest case are included in the equations of the gravitational field as a sum of quadratic constants; these constant terms, in a certain sense, replace the Einstein cosmological term, which ensures the presence of some repulsive forces. The overall effect is that at a certain value of its three-dimensional radius (depending on the magnitude of the torsion components), the model of a closed FRWL universe stops collapsing into a singularity and begins to re-expand, which allows us to associate the presence of a torsion variable with the presence of repulsive forces opposing gravitational attraction. This constitutes a new approach to the singularity problem.

The resonance from the discovery of the *torsion elimination of the cosmological singularity* among theorists in the 1970s was so great that within a short time hundreds of works appeared developing the gravitational theory of the spin-torsion interaction. The most popular among others was the so-called Einstein–Cartan–Kibble–Sciama theory in which the equations of the gravitational and torsion fields follow from the simplest Lagrangian, the density of the scalar curvature of space-time with the metric affine connection (cf. [90,91]). However, the general dissatisfaction with the limited possibilities of torsion in the Einstein-Cartan theory led to the appearance in the 1970–80s of many versions of torsion theories, in which torsion could propagate in a vacuum and was not always associated with the spin of the gravity source. Many of these theories were no longer purely ‘gravitational’, since, in addition to the gravitational constant, new coupling constants were introduced into the corresponding lagrangians.

Besides, numerous attempts were made to include geometric torsion components in the equations of electrodynamics (e.g. [92]), and also to link hypothetical spin-torsion effects with the properties of vacuum and the evolution of individual astrophysical objects (e.g. [93–95]).

The ideas of spin-torsion coupling can still be traced in current publications, and indeed in the last 10 years or so there has been an explosion of interest in extensions of the original torsion gravity theories under the name of *f(T) gravity* (see [96], §3.5.1, [97] for recent reviews). These theories have the advantage of having equations of second order, and they may account for dark energy and the late acceleration [98,99], but their relation to general relativity is not yet clear [100,101].

### (c) Singularity theorems

The definition of a singularity in cosmology as a ‘place’ where infinities appear has the difficulty that it may be made to disappear by some kind of ‘surgery’, removing the ‘bad’ part and ending up with a spacetime without singularities. The existence of singularities was not clearly proved until the pioneering work of C.W. Misner [102], R. Penrose, S. W. Hawking, R. Geroch and others in the period 1963–70 came along.

Penrose in 1965 [103] was the first to establish a black hole *singularity theorem* to the effect that given certain plausible assumptions about the validity of general relativity, the structure of spacetime, and the properties of matter fields, spacetime would be geodesically incomplete. Shortly after, Hawking adopted the topological methods introduced by Penrose and he proved several theorems for cosmological spacetimes [104], eventually leading to an ‘all-purpose’ singularity theorem in 1970 proved jointly by Hawking and Penrose [105].

These theorems, together with related work by Geroch [106,107] (see also the lectures [108–110], and the standard treatise [11]) demonstrated once and for all the supremacy of the use of topological methods in studying the global structure of spacetime. They also set a firm basis for the consideration of *the singularity problem*, which as emphasized by L. D. Landau, is one of the most important unsolved problems in theoretical physics [111,112].

### (d) BKL conjecture

During the same period, V. A. Belinski together with E. M. Lifshitz and I. M. Khalatnikov (BKL) came up with a general scheme about the asymptotic nature of spacetime and fields as one approaches the predicted generic singularity of the singularity theorems. This is a second aspect of the singularity problem, *the nature of the cosmological singularities*. Their landmark work led to the formulation of the *BKL conjecture*, a statement about the behaviour near the generic cosmological singularity. The resulting BKL behaviour is essentially of a new and eminently complicated type. No power-law behaviour may be ascribed to it, and at *each spatial point* on approach to the past singularity we have a situation described using a generalized Kasner solution and a subsequent homogeneous ‘Bianchi-type IX’ oscillatory behaviour, having an endless sequence of Kasner epochs grouped into eras all the way back to the beginning, cf. [113]. Misner [114] independently discovered this evolution in hamiltonian variables in the homogeneous case, and coined the resulting cosmology *the Mixmaster universe*.

Now in the general inhomogeneous case, the BKL conjecture has it that each spatial point, although evolving like one of these separate Bianchi IX homogeneous universes, experiences the collective gravitational wave perturbations of neighbouring spatial points, and the generic behaviour is such that almost all solutions of the Einstein equations approach an initial spacelike, vacuum-dominated, local and oscillatory singularity, [115].

However, in the case of inclusion of matter fields, in addition to the oscillatory behaviour of the solutions predicted by the BKL conjecture, there is a milder, monotonic approach to the big bang singularity, a type of local Kasner regime, namely, the asymptotic velocity term dominated (AVTD) behaviour, which is absent in the vacuum case. In this behaviour, no terms can grow exponentially and spatial derivative terms become negligible asymptotically, for instance as in the case of a scalar field [74].

Although the BKL conjecture is unproved until today, there are various different approaches to it. One approach to the general problem rewrites the system of equations into a form that directly generalizes from the spatially homogeneous case and formulates the BKL conjecture in this framework, cf. [68]. Another approach uses analytical and numerical techniques to support the validity of the BKL conjecture [116–120]. Still another approach to this basic problem is using new hamiltonian variables to reformulate the conjecture in a language suitable for M-theoretic cosmology, see section on M-theory in Part B of this survey.

### (e) Chaotic cosmology and horizons

One is therefore confronted with the general question of whether our universe evolves from a generic, possibly chaotic, initial state to the present one characterized by a large-scale order and homogeneity, or the other way around—from an orderly initial state to ‘chaos’.

Misner’s *chaotic cosmology program* [121,122] is based on the hamiltonian approach to the field equations of general relativity [123]. He thought to show that quite independently of any initial conditions in the early universe, more precisely if the universe was originally Mixmaster, the long-term future evolution will generically be characterized by a smooth and isotropic late universe, like the one we observe around us. Misner’s noble hope was that due to processes like those of frictional dissipation, irregularities in the initial distribution would have been erased very early, leaving an isotropic universe: ‘⋯the emphasis was on the refocusing of cosmological theory from measuring the FRWL constants to explaining why we live in an FRWL universe⋯’ [124]. For simple Bianchi types this is indeed true, but later it was shown that such smoothing processes were probably not efficient enough [125].

In fact, the problem of explaining why the universe is homogeneous on the largest scales must be decided long before the universe became Friedmann-like, due to a geometrical property of the non-intersection of the past light cones called *the horizon problem*. The existence of particle horizons would prevent the further synchronization of conditions in such a universe because domains in the microwave sky separated by more than a few degrees are causally disconnected, and, in fact, were so since the time of emission (last scattering surface). The Mixmaster universe, although describing a period before the Friedmannian stage of evolution of the universe, cannot solve the horizon problem and allow causal communication between such regions. This follows from detailed calculations of D. M. Chitre (cf. [126], chap. V), resulting in the probability for the vanishing of a horizon in the model to be 0.02%.

However, the Mixmaster universe in the Misner or the BKL formulations is clearly a chaotic system as it is demonstrated by a combination of numerical and analytic evidence [127–132] and references therein. This property is what makes the investigation of the generic inhomogeneous Mixmaster universe so difficult. Nowadays, the Mixmaster universe is studied using qualitative methods and the theory of attractors [133–135].

Therefore, the original hopes for explanation of the large-scale homogeneity through the homogeneous Mixmaster model did not work. Currently, the opposite direction is often cited as popular, the ‘order leading to chaos’ point of view is generally preferred, cf. [2], p. 212. A concrete suggestion for a ‘quiescent cosmology’, that is a universe with an isotropic initial singularity instead of a chaotic initial state, was made already in 1978 by J. D. Barrow in [136], and subsequently studied in detail by many workers in the field, cf. e.g. [137–139], and references therein.

### (f) The finite-action conjecture

The studies related to possible modifications of general relativity in conjunction with the works on the existence and nature of cosmological singularities in the 1960s led to doubts as to the general validity of many of the previous conclusions in cosmological models, and in fact to a host of new and unexplored directions of research.

A unique characteristic of these early developments was the introduction of techniques from global differential geometry, topology and dynamical systems into the field of general relativity. The problem of the *existence* of cosmological singularities was adequately solved by the singularity theorems in general relativity, but *the nature of singularities* remains a central problem in relativistic cosmology until today.

However, many are convinced that the predicted singularities in all important cases are accompanied by infinities in the curvatures and the thermodynamic properties of matter, and therefore at the singularities general relativity breaks down beyond repair. Thus, many feel that the proved existence of spacetime singularities in general relativity does not really point to a new and worthwhile physical effect that could be fruitfully further explored within general relativity theory (e.g. ‘does the singularity resemble something like a hydrodynamical shock wave rather than something more serious?’—cf. [140]).

Rather, the consensus seems to be that there is new physics operating at a level beyond general relativity and the standard model of cosmology, cf. e.g. [11,141,142]. Then, the singularity problem becomes only part of a more general (and complex!) *issue of the initial conditions* for the early universe. This is so because one of the assumptions leading to the existence of singularities is that of the general validity of general relativity and that assumption becomes invalid when considered in the framework of possible *geometric extensions of general relativity*, or of the still unknown theory *quantum gravity* or, more generally, that of the still unfinished *unified theory of all fundamental interactions*. In that sense, one may be allowed or even required to consider general approaches of the cosmological problem in all of these contexts, and this opens up a huge field of further investigation.

However, it is important to point out that the situation may not be so clear-cut as one would naively expect. This is so because there is a correlation between the lack of spacetime singularities in the curvature in general relativity or some future fundamental theory, and singularities that will by necessity appear in the universal action of the theory. Indeed, the *finite-action conjecture* [143–145] relates to exactly that the finiteness of the universal action of some theory applicable to cosmology depends on the existence of past and future spacetime curvature singularities because that finiteness results from an integration over finite time intervals. When action singularities are avoided then new spacetime singularities will arise and *vice versa*, when spacetime singularities are absent in a theory then new action singularities will emerge. Another important point is that since paths of finite actions have generally zero-measure contributions to the path integral for many examples such as the simple harmonic oscillator [146], the correct path integral in cosmology must be such that such finite action contributions become the dominant ones, and this might be a very restrictive requirement. This is a frontier of modern cosmological research.

## 4. Contents and abstracts of the theme issue, Volume 1

Theoretical mathematical cosmology, a most majestic of fields, is not a subject that an interested person may easily enter. When one overcomes the difficulties of acquiring a proper or needed background, one will need a clear compass as to what research problems and directions are available, important or doable.

In previous sections, we have given some taste of the amazing variety of research in this field by discussing some of the most important models, theories and remaining problems in the field. We have focused on various cosmological models, such as the Einstein static universe, de Sitter space, Lemaître universe, Einstein-de Sitter universe, Tolman’s oscillation model, the Friedmann family of cosmologies, the Kasner universe, the Lemaître-Tolman inhomogeneous model, Lifshitz perturbed universes, Gödel spacetime, and many other universes.

We have given short descriptions of various cosmological theories such as the hot big bang, the steady-state theory, chaotic cosmology, inflation, the multiverse, quantum universes, Kaluza–Klein and other multi-dimensional worlds, stringy universes, braneworlds and the landscape. These theories in turn use for their formulations physical theories that aim at describing gravitation at various stages of the evolution of the universe, from general relativity, to modified gravity, to string and M-theory and to dark energy.

However, many theoretical challenges remain. The singularity problem is one of them, but we have also included discussions of the BKL conjecture, the measure problem, the stability issue, topological problems, the classification and nature of dynamical singularities, the horizon, flatness and entropy problems, and the problem of genericity in cosmology.

Research today in mathematical cosmology is active in all these aspects of the field, while it appears that research papers in mathematical cosmology are scattered in diverse publications, together with other, often very different research, and sometimes it becomes easy to lose orientation. We therefore end this paper with the inclusion of the abstracts of all contributions to the theme issue ‘The Future of Mathematical Cosmology’, in an effort to give the reader some idea as to where research in mathematical and theoretical cosmology will be heading today and in the coming years.

Many of the invited contributors of this theme issue are leaders of research in this area. We believe that their collective presence in this volume will provide a direct proof of the richness and importance of the whole field, as well as the required orientation, without further need of defence. We hope that the scientific community will be interested to see what is included in this collection of papers, the titles and abstracts of which are reproduced in this section (reference numbers in these abstracts correspond to those in the specific papers), hoping that this theme issue may become a unique pole of attraction and a point of reference for future developments.

This theme issue contains many of the important results and meaningful research directions of the future, their connections to the past, as well as interconnections to other independent but important issues of the same field, that researchers may not easily have at their disposal or discover by themselves. Works appearing here have a direct relevance to the various topics touched on in this paper, and we hope their diverse nature will look somewhat more coherent by including these abstracts here.

Interest in theoretical mathematical cosmology was recently renewed by the decision to award the 2021 Nobel Prize in Physics to R. Penrose for his work on the proof of the first singularity theorem in 1965, a result which appears as one of the central pillars of the whole field of modern mathematical cosmology as we showed in the previous sections. This theme issue appearing in the Philosophical Transactions of the Royal Society A as an edited collection of important results, problems and future research directions in this field will, we therefore hope, influence the wider scientific community as well as policy makers to further appreciate its unique importance for the future development of this fundamental mathematical science, and theoretical cosmology.

### (a) Volume 1

The abstract of the contributions to Volume 1 of the theme issue are as follows.

(i) S. Scott and P. Threlfall, Cosmological Milestones, Conformal Frameworks and Quiescent Cosmology
  To understand the nature of the birth of our Universe and its eventual demise is a driving force in theoretical physics and, indeed, for humanity. A zoo of definitions has appeared in the literature to catalogue different types of cosmological milestones such as ‘Big Bangs’, ‘Big Crunches’, ‘Big Rips’, ‘Sudden Singularities’, ‘Bounces’ and ‘Turnarounds’. Quiescent cosmology is the notion that the Universe commenced in a Big Bang that was highly regular and smooth and evolved away from this initial isotropy and homogeneity due to gravitational attraction. The quiescent cosmology concept meshes well with Penrose’s ideas regarding gravitational entropy and the clumping of matter, and the associated Weyl Curvature Hypothesis. Conformal frameworks, such as the Isotropic Past Singularity, have been devised to encapsulate initial and final states for the Universe which are in accordance with these programs. Since much of the research on cosmological milestones has been focussed on FRW solutions, many of which possess initial singularities which are isotropic Big Bangs, we analyse here the relationship between cosmological milestones and conformal frameworks for these solutions. We establish the general properties of FRW models which admit these conformal frameworks, including whether they satisfy various energy conditions, and are therefore physically reasonable.
(ii) V. Moncrief and P. Mondal, Einstein flow with matter sources: stability and convergence
  Two recent articles [1, 2] suggested an interesting dynamical mechanism within the framework of the vacuum Einstein flow (or Einstein-Λ flow if a positive cosmological constant Λ is included) which suggests that many closed (compact without boundary) manifolds that do not support homogeneous and isotropic metrics at all will nevertheless evolve to be asymptotically compatible with the observed approximate homogeneity and isotropy of the physical universe. These studies however did not include matter sources. Therefore, the aim of the present study is to include suitable matter sources and investigate whether one is able to draw a similar conclusion.
(iii) E. Ames, F. Beyer, J. Isenberg, and T. A. Oliynyk, Stability of Asymptotic Behavior Within Polarised T2- Symmetric Vacuum Solutions with Cosmological Constant
  We prove the nonlinear stability of the asymptotic behaviour of perturbations of subfamilies of Kasner solutions in the contracting time direction within the class of polarized $T^{2}$-symmetric solutions of the vacuum Einstein equations with arbitrary cosmological constant Λ. This stability result generalizes the results proven in [3], which focus on the Λ=0 case, and as in that article, the proof relies on an areal time foliation and Fuchsian techniques. Even for Λ=0, the results established here apply to a wider class of perturbations of Kasner solutions within the family of polarized $T^{2}$-symmetric vacuum solutions than those considered in [3] and [26]. Our results establish that the areal time coordinate takes all values in $\left(0,T_{0}\right]$ for some $T_{0}>0$, for certain families of polarized T2-symmetric solutions with cosmological constant.
(iv) J.M.M. Senovilla, A critical appraisal of the singularity theorems
  The 2020 Nobel Prize in Physics has revived the interest in the singularity theorems and, in particular, in the Penrose theorem published in 1965. In this short paper, I briefly review the main ideas behind the theorems and then proceed to an evaluation of their hypotheses and implications. I will try to dispel some common misconceptions about the theorems and their conclusions, as well as to convey some of their rarely mentioned consequences. In particular, a discussion of spacetime extensions in relation to the theorems is provided. The nature of the singularity inside black holes is also analysed.
(v) T. Padmanabhan, Lessons from the cosmological constant about the nature of gravity
  Brief description: The existence of a positive cosmological constant allows us to infer several key features about the true nature of gravity. I will describe how this approach demands a paradigm shift in our description of gravity and suggests that gravity is an emergent phenomenon.
  *Editors’ Comment: It was with great sorrow that we learnt that Thanu Padmanabhan passed away on September 17 September 2021, a few days before he was to submit his invited contribution to this theme issue. We very much regret the loss of Paddy, a great cosmologist and an amazing human being. We include here the original title and abstract of his contribution, which he sent to us in an email on 18 February 2021.*
(vi) K. A. Bronnikov, Some unusual wormholes in general relativity
  In this short review we present some recently obtained traversable wormhole models in the framework of general relativity (GR) in four and six dimensions that somehow widen our common ideas on wormhole existence and properties. These are, first, rotating cylindrical wormholes, asymptotically flat in the radial direction and existing without exotic matter. The topological censorship theorems are not violated due to lack of asymptotic flatness in all spatial directions. Second, these are cosmological wormholes constructed on the basis of the Lemaitre–Tolman–Bondi solution. They connect two copies of a closed Friedmann world filled with dust, or two otherwise distant parts of the same Friedmann world. Third, these are wormholes obtained in six-dimensional GR, whose one entrance is located in ‘our’ asymptotically flat world with very small extra dimensions while the other ‘end’ belongs to a universe with large extra dimensions and therefore different physical properties. The possible observable features of such wormholes are briefly discussed.
(vii) S. Cotsakis, Onset of synchronization in coupled Mixmaster oscillators
  We consider the problem of asymptotic synchronization of different spatial points coupled to each other in inhomogeneous spacetime and undergoing chaotic Mixmaster oscillations towards the singularity. We demonstrate that for couplings larger than some threshold value, two Mixmaster spatial points A,B, with A in the past of B, synchronize and thereby proceed in perfect unison towards the initial singularity. We further show that there is a Lyapunov function for the synchronization dynamics that makes different spatial points able to synchronize exponentially fast in the past direction. We provide an elementary proof of how an arbitrary spatial point responds to the mean field created by the oscillators, leading to their direct interaction through spontaneous synchronization. These results ascribe a clear physical meaning of early-time synchronization leading to a resetting effect for the two BKL maps corresponding to two distinct oscillating spatial points, as the two maps converge to each other to become indistinguishable at the end of synchronization. Our results imply that the universe generically organizes itself through simpler, synchronized, states as it approaches the initial singularity. A discussion of further implications of early-time inhomogeneous Mixmaster synchronization is also provided.
(viii) Ruth Lazkoz and Leonardo Fernández–Jambrina, New futures for cosmological models
  The discovery of accelerated expansion of the universe opened the possibility of new scenarios for the doom of our spacetime, besides aeternal expansion and a final contraction. In this paper we review the chances which may await our universe. In particular, there are new possible singular fates (sudden singularities, big rip⋯), but there also other evolutions which cannot be considered as singular. In addition to this, some of the singular fates are not strong enough in the sense that the spacetime can be extended beyond the singularity. For deriving our results, we make use of generalized power expansions of the scale factor of the universe.
(ix) V. Ivashchuk, On stable exponential cosmological solutions with two factor spaces in (1+m+2)-dimensional EGB model with Λ-term
  A (m+3)-dimensional Einstein–Gauss–Bonnet gravitational model including the Gauss–Bonnet term and the cosmological term Λ is considered. Exact solutions with exponential time dependence of two scale factors, governed by two Hubble-like parameters H>0 and h≠H, corresponding to factor spaces of dimensions m>2 and l=2, respectively, are found. Under certain restrictions on x=h/H, the stability of the solutions in a class of cosmological solutions with diagonal metrics is proved. A subclass of solutions with small enough variation of the effective gravitational constant G is considered and the stability of all solutions from this subclass is shown.
(x) N. Mavromatos, Geometrical origins of the Universe dark sector: string-inspired torsion and anomalies as seeds for inflation and dark matter
  In a modest attempt to present potentially new paradigms in cosmology, including its inflationary epoch, and initiate discussions, I review in this article some novel, string-inspired cosmological models, which entail a purely geometrical origin of the dark sector of the Universe but also of its observed matter-antimatter asymmetry. The models contain gravitational (string-model independent, Kalb–Ramond (KR)) axion fields coupled to primordial gravitational anomalies via CP-violating interactions. The anomaly terms are four-space-time-dimensional remnants of the Green-Schwarz counterterms appearing in the definition of the field strength of the spin-one antisymmetric tensor field of the (bosonic) massless gravitational string multiplet, which also plays the role of a totally antisymmetric component of torsion. I show how in such cosmologies the presence of primordial gravitational waves can lead to anomaly condensates and dynamical inflation of a ‘running vacuum-model’ type, without external inflatons, but also to leptogenesis in the radiation era due to anomaly-induced Lorentz and CPT Violating KR axion backgrounds. I also discuss how the torsion-related KR-axion could acquire a mass during the QCD epoch, thus playing the role of (a component of) Dark Matter. Phenomenological considerations of the inflationary and post-inflationary (in particular, modern) eras of the model are briefly discussed, including its potential for alleviating the observed tensions in the cosmological data of the current epoch.
(xi) G. Horndeski, Reformulating scalar-tensor field theories as scalar-scalar field theories using a novel geometry
  In this paper I shall show how the notions of Finsler geometry can be used to construct a similar type of geometry using a scalar field, f, on the cotangent bundle of a differentiable manifold, M. This will enable me to use the second vertical derivatives of f, along with the differential of a scalar field ϕ on M, to construct a Lorentzian metric tensor on M that depends upon ϕ. I refer to a field theory based upon a manifold with such a Lorentzian structure as a scalar-scalar field theory. We shall study such a theory when f is chosen so that the resultant metric on M has the form of a Friedman–Lemaitre–Robertson–Walker metric, with the t equal constant slices being flat, and ϕ being a function of t. When second-order scalar-tensor Lagrangians are evaluated for this choice of geometry, they give rise to Lagrangians which are functions only of ϕ and its time derivatives. I refer to these ‘scalarized’ Lagrangians as ‘hidden Lagrangians’. However, not all Lagrangians used in scalar-scalar field theories need come from scalar-tensor Lagrangians. A particularly simple ‘pure’ scalar-scalar Lagrangian will be investigated in detail. It will be shown that this Lagrangian can generate self-inflating universes, which can be pieced together to form multiverses with non-Hausdorff topologies in which the global time function multifurcates at t=0. Some of the universes in these multiverses begin explosively, and then settle down to a period of much quieter accelerated expansion, which can be followed by a collapse to their original, pre-expansion state.

## Data Availability

This article has no additional data.

## Footnotes

1 For reprints of early papers appearing in the text for the next few subsections, see [6]. For an early history of theoretical cosmology in the 20th century, see [7].

## References

[1] Landau LD, Lifshitz EM. 1977 Quantum mechanics, 3rd edn. Oxford, UK: Pergamon Press.

[2] Peebles PJE. 2020 Cosmology’s century. Princeton, NJ: Princeton University Press.

[3] Ellis GFR, Maartens R, MacCallum MAH. 2012 Relativistic cosmology. Cambridge, UK: CUP.

[4] Cotsakis S. 2002 Astrophys. Space Sci. Libr., 276, p. 5. e-Print: gr-qc/0107090 [gr-qc]; Lect. Notes Phys. 592 (2002) 59–94, e-Print: gr-qc/0201067.

[5] Peter P, Uzan J-P. 2009 Primordial cosmology. New York, NY: OUP.

[6] Bernstein J, Feinberg G (eds). 1989 Cosmological constants: papers in modern cosmology. New York, NY: Columbia University Press.

[7] North JD. 1965 The measure of the universe: a history of modern cosmology. New York, NY: Dover.

[8] Einstein A. 1917 Cosmological considerations in the general theory of relativity. Sitzungsber. Preuss. Akad. Wiss, Berlin (Math. Phys.) **1917**, 142-152. English translation in The Principle of Relativity (eds HA Lorentz, A Einstein, H Minkowski, H Weyl). Dover, 1923.

[9] De Sitter W. 1917 Einstein’s theory of gravitation and its astronomical consequences. Mon. Not. R. Astron. Soc. **78**, 3-28. (10.1093/mnras/78.1.3)

[10] Rindler W. 2006 Relativity: special, general, and cosmological, 2nd edn. Oxford, UK: OUP.

[11] Hawking SW, Ellis GFR. 1973 The large-scale structure of space-time. Cambridge, UK: CUP.

[12] Gibbons GW, Hawking SW. 1977 Cosmological event horizons, thermodynamics, and particle creation. Phys. Rev. D **15**, 2738-2751. (10.1103/PhysRevD.15.2738)

[13] Perry MJ. 1983 An instability of De Sitter space. In *The very early universe*. Proceedings of the Nuffield Workshop, Cambridge, 21 June to 9 July, 1982, (eds GW Gibbons, SW Hawking, STC Siklos). CUP.

[14] Charlier C. 1922 Arkiv. f. Matematik och Fysik, 16, no. 2.

[15] Mandelbrot BB. 1975 Sur un modèle décomposable d'univers hiérarchisé: déduction des corrélations galactiques sur la sphère céleste. C. R. Acad. Sci. (Paris) **A280**, 1551.

[16] Harrison E. 2000 Cosmology: the science of the universe. Cambridge, UK: CUP.

[17] Arnol’d VI. 1990 Huygens and Barrow, Newton and Hooke. Berlin, Germany: Birkhäuser. Par. 5; *Mathematical methods of classical mechanics*, 2nd Ed. New York, NY: Springer, 1989. App. 15.

[18] Selety F. 1922 Beiträge zum kosmologischen Problem. Annalen der Physik **68**, 281-334. (10.1002/andp.19223731202)

[19] de Vaucoleurs G. 2000 The case for a hierarchical cosmology. Science **167**, 1203-1213. (10.1126/science.167.3922.1203) 17751407

[20] Dyer C. 1979 A spherically symmetric self-similar universe. Mon. Not. R. Astron. Soc. **189**, 189-201. (10.1093/mnras/189.2.189)

[21] Labini FS, Montuori M, Pietronero L. 1998 Scale invariance of galaxy clustering. Phys. Rept. **293**, 61-226. (10.1016/S0370-1573(97)00044-6)

[22] Labini FS. 2010 Characterizing the large scale inhomogeneity of the galaxy distribution. In *AIP Conf. Proc.*, vol. 1241, pp. 981–990. e-Print: 0910.3833 [astro-ph.CO].

[23] De Marzo G, Labini FS, Pietronero L. 2021 Zipf’s law for cosmic structures: how large are the greatest structures in the universe. Astron. Astrophys. **651**, A114. (10.1051/0004-6361/202141081)

[24] Friedmann A. 1922 Über die krümmung des raumes. Z. Phys. **10**, 377-386. (10.1007/BF01332580)

[25] Friedmann A. 1924 Über die Möglichkeit einer Welt mit konstanter negativer Krümmung des Raumes. Z. Phys. **21**, 326-332. (10.1007/BF01328280)

[26] Slipher VM. 1915 Spectrographic observations of nebulae. Pop. Astron. **23**, 21-24.

[27] Lemaître G. 1927 Un Univers homogène de masse constante et de rayon croissant rendant compte de la vitesse radiale des nébuleuses extra-galactiques. Ann. Soc. Sci. Brux. **A47**, 49-59.

[28] Lemaître G. 1931 Expansion of the universe, A homogeneous universe of constant mass and increasing radius accounting for the radial velocity of extra-galactic nebulae. Mon. Not. R. Astron. Soc. **91**, 483-490. (10.1093/mnras/91.5.483)

[29] Barrow JD. 2011 The book of universes. London, UK: Bodley Head.

[30] Eddington AS. 1930 On the instability of Einstein’s spherical world. Mon. Not. R. Astron. Soc. **90**, 668-678. (10.1093/mnras/90.7.668)

[31] Lemaître G. 1934 Evolution of the expanding universe. Proc. Natl Acad. Sci. USA **20**, 12-17. (10.1073/pnas.20.1.12) 16587831 PMC1076329

[32] Einstein A, de Sitter W. 1932 On the relation between the expansion and the mean density of the universe. Proc. Natl Acad. Sci. USA **18**, 213-214. (10.1073/pnas.18.3.213) 16587663 PMC1076193

[33] Tolman R. 1934 Relativity, thermodynamics and cosmology. New York, NY: Dover.

[34] Kasner E. 1921 Geometrical theorems on Einstein’s cosmological equations. Am. J. Math. **43**, 217-221. (10.2307/2370192)

[35] Lemaître G. 1933 Generalization of the de Sitter Cosmos. Ann. Soc. Sci. Bruxelles A **53**, 51-85. English transl. (1997). Gen. Rel. Grav. 29, 641.

[36] Tolman RC. 1934 Effect of inhomogeneity on cosmological models. Proc. Natl Acad. Sci. USA **20**, 169-176. repinted (1997). Gen. Rel. Grav. 29, 935. (10.1073/pnas.20.3.169) 16587869 PMC1076370

[37] Einstein A, Straus EG. 1945 The influence of the expansion of space on the gravitation fields surrounding the individual stars. Rev. Mod. Phys. **17**, 120. *ibid* 18 (1946) 148. (10.1103/RevModPhys.17.120)

[38] Einstein A, Rosen N. 1935 The particle problem in the general theory of relativity. Phys. Rev. **48**, 73. (10.1103/PhysRev.48.73)

[39] Capovilla R, Guven J. 1995 Large deformations of relativistic membranes: a generalization of the Raychaudhuri equations. Phys. Rev. D **52**, 1072. 10.1103/physrevd.52.1072 10019324

[40] Carter B. 2001 Essentials of classical Brane dynamics. Int. J. Theor. Phys. **40**, 2099-2130. e-Print: gr-qc/0012036 [gr-qc]. (10.1023/A:1012934901706)

[41] Mannheim PD. 2005 Brane-localized gravity. Singapore: World Scientific.

[42] Lifshitz E. 1946 Republication of: On the gravitational stability of the expanding universe. J. Phys. U.S.S.R. **10**, 116.

[43] Belinski VA, Khalatnikov IM, Lifshitz EM. 1963 Investigations in relativistic cosmology. Adv. Phys. **12**, 185-249. (10.1080/00018736300101283)

[44] Landau LD, Lifshitz EM. 1975 The classical theory of fields, 4th Rev. edn. Oxford, UK: Pergamon Press.

[45] For recent work, see Tomita K. 2005 Relativistic second-order perturbations of nonzero-Λ flat cosmological models and CMB anisotropies. Phys. Rev. D **71**, 3504. (10.1103/PhysRevD.71.083504)

[46] Bartolo N, Matarrese S, Riotto A. 2006 Cosmic microwave background anisotropies up to second order. J. Cosm. Astropart. Phys. **0606**, 024. (10.1088/1475-7516/2006/06/024)

[47] Weinberg SW. 2007 Cosmology. New York, NY: OUP.

[48] Gamow G. 1948 The evolution of the universe. Nature **162**, 680. (10.1038/162680a0) 18893719

[49] Gamow G. 1952 The creation of the universe. New York: Viking Press.

[50] Alpher RA, Herman RC. 1990 *Early work on the ‘big-bang’ cosmology and the microwave background radiation*. In *Modern Cosmology in Retrospect* (eds B Bertotti *et al.*) CUP.

[51] Weinberg SW. 1993 The first three minutes: a modern view of the origin of the universe, 2nd edn. New York, NY: Basic Books.

[52] Bondi H, Gold T. 1948 The steady-state theory of the expanding universe. Mon. Not. R. Astron. Soc. **108**, 252-270. (10.1093/mnras/108.3.252)

[53] Hoyle F. 1948 A new model for the expanding universe. Mon. Not. R. Astron. Soc. **108**, 342.

[54] Narlikar JV. 1983 Introduction to cosmology. Boston, MA: Jones & Bartlett.

[55] McCrea WH. 1951 Relativity theory and the creation of matter. Proc. R. Soc. **206**, 562.

[56] McCrea WH. 1964 Continual creation. Mon. Not. R. Astron. Soc. **128**, 335-344. (10.1093/mnras/128.4.335)

[57] Penrose R. 2010 Cycles of time. London, UK: Bodley Head.

[58] Gödel K. 1949 An example of a new type of cosmological solutions of Einstein’s field equations of gravitation. Rev. Mod. Phys. **21**, 447. (10.1103/RevModPhys.21.447)

[59] Tipler FJ, Clarke CJS, Ellis GFR. 1980 Singularities and horizons: a review article. In *General Relativity and Gravitation: One Hundred Years after the Birth of Albert Einstein* (ed. A Held), vol. 2, p. 97. Pleanum.

[60] Earman J. 1995 Bangs, Crunches, Whimpers, and Shrieks. Oxford: OUP.

[61] Reboucas MJ, Tiomno J. 1983 Homogeneity of Riemannian space-times of Gödel type. Phys. Rev. D **28**, 1251. (10.1103/PhysRevD.28.1251)

[62] Accioly AJ. 1987 An unusual cosmological solution in the context of higher-derivative gravity. Nuovo Cimento **B100**, 703.

[63] Barrow JD, Dabrowski MP. 1998 Gödel universes in string theory. Phys. Rev. D **58**, 103502. (10.1103/PhysRevD.58.103502)

[64] Boyda EK, Ganguli S, Horava P, Varadarajan U. 2003 Holographic protection of chronology in universes of the Gödel type. Phys. Rev. D **67**, 106003. (10.1103/PhysRevD.67.106003)

[65] Taub AH. 1951 Empty space-times admitting a three parameter group of motions. Ann. Math. **53**, 472-490. (10.2307/1969567)

[66] Bianchi L. 1897 Mem. Soc. It. della. Sc. (Dei. XL) (3) **11**, 267.

[67] Krasinski A. 1997 Inhomogeneous cosmological models. Cambridge, UK: CUP.

[68] Uggla C, van Elst H, Wainwright J, Ellis GFR. 2003 Past attractor in inhomogeneous cosmology. Phys. Rev. D **68**, 103502. (10.1103/PhysRevD.68.103502)

[69] Dirac PAM. 1937 The cosmological constants. Nature **139**, 323. (10.1038/139323a0)

[70] Brans C, Dicke RH. 1961 Mach’s principle and a relativistic theory of gravitation. Phys. Rev. **124**, 925. (10.1103/PhysRev.124.925)

[71] Dicke RH. 1968 The theoretical significance of experimental relativity. New York, NY: Gordon and Breach.

[72] Nariai H. 1969 Gravitational instability in the Brans-Dicke cosmology. Prog. Theo. Phys. **42**, 544-554. (10.1143/PTP.42.544)

[73] Ruban VA, Filkenstein AM. 1975 On the initial singularity in the scalar-tensor anisotropic cosmology. Gen. Rel. Grav. **6**, 601. (10.1007/BF00761966)

[74] Belinskii VA, Khalatnikov IM. 1973 Effect of scalar and vector fields on the nature of the cosmological singularity. Sov. Phys. JETP **36**, 591-597.

[75] Ruban VA. 1977 On the influence of massless scalar and vector electromagnetic fields on the singularity character in anisotropic cosmology. Astroph. Space Sci. **46**, L23. (10.1007/BF00644398)

[76] Kolitch SJ, Eardley DM. 1995 Behavior of Friedmann-Robertson-Walker cosmological models in scalar-tensor gravity. Ann. Phys. **241**, 128-151. (10.1006/aphy.1995.1058)

[77] Brans CH. Gravity and the tenacious scalar field. In *Symposium to honor Engelbert Schucking*. e-Print: gr-qc/9705069 [gr-qc].

[78] Brans CH. 2004 *The Roots of scalar-tensor theory: An approximate history*. In the: Santa Clara 2004: 1st International Workshop on Gravitation and Cosmology. e-Print: gr-qc/0506063 [gr-qc].

[79] Dicke RH. 1962 Mach’s principle and invariance under transformation of units. Phys. Rev. **125**, 2163. (10.1103/PhysRev.125.2163)

[80] Barrow JD. 2003 Constants and variations: from alpha to omega. Astrophys. Space Sci. **283**, 645-660. (10.1023/A:1022571927342)

[81] Barrow JD. 2002 The constants of nature: from alpha to omega. London, UK: Jonathan Cape.

[82] Damour T, Nordtvedt K. 1993 Tensor-scalar cosmological models and their relaxation toward general relativity. Phys. Rev. D **48**, 3436-3450. (10.1103/PhysRevD.48.3436) 10016613

[83] Mimoso JP, Nunes AM. 1998 General relativity as a cosmological attractor of scalar-tensor gravity theories. Phys. Lett. A **248**, 325-331. (10.1016/S0375-9601(98)00724-5)

[84] Gasperini M. 2007 Elements of string cosmology. Cambridge, UK: CUP.

[85] Cartan E. 1924 Sur les variétés à connexion affine, et la théorie de la relativité généralisée. Annales scientifiques de l’E.N.S. 3e série, tome **41**, 1-0.

[86] Kibble TWB. 1961 Lorentz invariance and the gravitational field. J. Math. Phys. **2**, 212-221. (10.1063/1.1703702)

[87] Sciama DW. 1961 The physical structure of general relativity. Rev. Mod. Phys. **36**, 463-469. (10.1103/RevModPhys.36.463)

[88] Kopczynski W. 1972 A non-singular universe with torsion. Phys. Lett. A **39**, 219-220. (10.1016/0375-9601(72)90714-1)

[89] Trautman A. 1973 Spin and torsion may avert gravitational singularities. Nature **242**, 7-8.

[90] Hehl FW, von der Heyde P, Kerlick GD. 1976 General relativity with spin and torsion: foundations and prospects. Rev. Mod. Phys. **48**, 393-416. (10.1103/RevModPhys.48.393)

[91] Hehl FW, McCrea JD, Mielke EW, Ne’eman Y. 1995 Metric-affine gauge theory of gravity: field equations, Noether identities, world spinors, and breaking of dilation invariance. Phys. Rept. **258**, 1-171. (10.1016/0370-1573(94)00111-F)

[92] de Sabbata V, Gasperini M. 1980 A ‘semi-minimal’ coupling principle for the electromagnetic field in a space with torsion. Lettere al Nuovo Cimento **28**, 229-233. (10.1007/BF02770214)

[93] de Sabbata V, Gasperini M. 1980 Torsion contributions to the magnetic field of a spinning charge. Lettere al Nuovo Cimento **28**, 234-236. (10.1007/BF02770215)

[94] de Sabbata V, Gasperini M. 1980 Spin alignment in a neutron star. Lettere al Nuovo Cimento **27**, 289-292. (10.1007/BF02817188)

[95] Yefremov AP. 1981 Configuration of a neutron star with axially polarized spin. Acta Phys. Pol. **3**, 185-188.

[96] Clifton T, Ferreira PG, Padilla A, Skordis C. 2012 Modified gravity and cosmology. Phys. Rept. **513**, 1-189. (10.1016/j.physrep.2012.01.001)

[97] Cai YF, Capozziello S, De Laurentis M, Saridakis EN. 2016 f (T) teleparallel gravity and cosmology. Rep. Prog. Phys. **79**, 106901. (10.1088/0034-4885/79/10/106901) 27599606

[98] Bengochea GR, Ferraro R. 2009 Dark torsion as the cosmic speed-up. Phys. Rev. D **79**, 124019. (10.1103/PhysRevD.79.124019)

[99] Linder EV. 2010 Einstein’s other gravity and the acceleration of the Universe. Phys. Rev. D **81**, 127301. *Phys. Rev. D* 82 (2010) 109902 (erratum). e-Print: 1005.3039 [astro-ph.CO]. (10.1103/PhysRevD.81.127301)

[100] Combi L, Romero GE. 2018 Is teleparallel gravity really equivalent to general relativity? Annalen Phys. **530**, 1700175.

[101] Yang R-J. 2011 Conformal transformation in f(T) theories. Europhys. Lett. **93**, 60001. (10.1209/0295-5075/93/60001)

[102] Misner CW. 1963 The Flatter Regions of Newman, Unti, and Tamburino’s Generalized Schwarzschild Space. J. Math. Phys. **7**, 924-937. (10.1063/1.1704019)

[103] Penrose R. 1965 Gravitational collapse and space-time singularities. Phys. Rev. Lett. **14**, 57. (10.1103/PhysRevLett.14.57)

[104] Hawking SW. 1966 The occurrence of singularities in cosmology. Proc. R. Soc. Lond. A **294**, 511. *ibid* 295 (1966) 490; *ibid* 300 (1967) 187. (10.1098/rspa.1966.0221)

[105] Hawking SW, Penrose R. 1970 The singularities of gravitational collapse and cosmology. Proc. R. Soc. Lond. A **314**, 529. (10.1098/rspa.1970.0021)

[106] Geroch R. 1968 What is a singularity in general relativity? Ann. Phys. **48**, 526-540. (10.1016/0003-4916(68)90144-9)

[107] Geroch R. 1970 Domain of dependence. J. Math. Phys. **11**, 437. (10.1063/1.1665157)

[108] Geroch R. 1971 General relativity in the large. Gen. Rel. Grav. **2**, 61-74. (10.1007/BF02450519)

[109] Ellis GFR. 1971 Topology and cosmology. Gen. Rel. Grav. **2**, 7-21. (10.1007/BF02450512)

[110] Penrose R. 1972 Techniques of differential topology in relativity. Philadelphia, PA: SIAM.

[111] Khalatnikov IM, Kamenshchik AYu. 2008 Conferences and Symposia: Lev Landau and the problem of singularities in cosmology. Phys. Usp. **51**, 609-616. (10.1070/PU2008v051n06ABEH006550)

[112] Belinski VA. 2014 On the cosmological singularity. Int. J. Mod. Phys. D **23**, 1430016. (10.1142/S021827181430016X)

[113] Belinski VA, Khalatnikov IM, Lifshitz EM. 1970 Oscillatory approach to a singular point in the relativistic cosmology. Adv. Phys. **19**, 525-573. (10.1080/00018737000101171)

[114] Misner CW. 1969 Mixmaster Universe. Phys. Rev. Lett. **22**, 1071. (10.1103/PhysRevLett.22.1071)

[115] Belinski VA, Khalatnikov IM, Lifshitz EM. 1982 A general solution of the Einstein equations with a time singularity. Adv. Phys. **31**, 639. (10.1080/00018738200101428)

[116] Berger BK, Moncrief V. 1993 Numerical investigation of cosmological singularities. Phys. Rev. D **48**, 4676. (10.1103/PhysRevD.48.4676) 10016121

[117] Berger BK, Moncrief V. 1998 Numerical evidence that the singularity in polarized U(1) symmetric cosmologies on $T^{3}\times R$ is velocity dominated. Phys. Rev. D **57**, 7235. (10.1103/PhysRevD.57.7235)

[118] Berger BK, Moncrief V. 1998 Evidence for an oscillatory singularity in generic U(1) symmetric cosmologies on $T^{3}\times R$. Phys. Rev. D **58**, 064023. (10.1103/PhysRevD.58.064023)

[119] Berger BK, Garfinkle D, Isenberg J, Moncrief V, Weaver M. 1998 The singularity in generic gravitational collapse is spacelike, local and oscillatory. Mod. Phys. Lett. A **13**, 1565-1573. (10.1142/S0217732398001649)

[120] Berger BK, Isenberg J, Weaver M. 2001 Oscillatory approach to the singularity in vacuum spacetimes with $T^{2}$ isometry. Phys. Rev. D **64**, 084006. (10.1103/PhysRevD.64.084006)

[121] Misner CW. 1968 The isotropy of the universe. Astrophys. J. **151**, 431-457. (10.1086/149448)

[122] Misner CW. 1969 Mixmaster universe. Phys. Rev. Lett. **22**, 1071-1074. (10.1103/PhysRevLett.22.1071)

[123] Arnowitt R, Deser S, Misner CW. 1962 The dynamics of general relativity. In *Gravitation: An Introduction to Current Research* (ed. L Witten), p. 227. New York: Wiley. reprinted in GEn Rel. Grav. 40 (2008) 1997.

[124] Misner CW. 1994 The Mixmaster cosmological metrics. In *Deterministic Chaos in General Relativity* (ed. D Hobill). Plenum Press.

[125] Barrow JD, Matzner RA. 1977 The homogeneity and isotropy of the Universe? Mon. Not. R. Astron. Soc. **181**, 719-727. (10.1093/mnras/181.4.719)

[126] Chitre DM. 1972 *Investigation of vanishing of a horizon for Bianchi type IX (the Mixmaster universe)*. PhD Thesis, Technical Report No. 72–125. Univ. of Maryland. Faculty Publications Collection.

[127] Barrow JD. 1982 Chaotic behaviour in general relativity. Phys. Rep. **85**, 1-49. (10.1016/0370-1573(82)90171-5)

[128] Barrow JD, Cotsakis S. 1989 Chaotic Behaviour in higher-order gravity theories. Phys. Lett. B **232**, 172-176. (10.1016/0370-2693(89)91681-X)

[129] Cotsakis S, Demaret J, de Rop Y, Querella L. 1993 Mixmaster universe in fourth-order gravity theories. Phys. Rev. D **48**, 4595. (10.1103/PhysRevD.48.4595) 10016110

[130] Hobill D, Bernstein D, Welge M, Simkins D. 1991 The Mixmaster cosmology as a dynamical system. Class. Quant. Grav. **8**, 1155. (10.1088/0264-9381/8/6/013)

[131] Berger BK. 1994 The BKL discrete evolution as an approximation to Mixmaster dynamics. In *Deterministic Chaos in General Relativity* (ed. D Hobill). Plenum Press.

[132] Demaret J, De Rop Y. 1993 The fractal nature of the power spectrum as an indicator of chaos in the Bianchi-IX cosmological model. Phys. Lett. B **299**, 223-228. (10.1016/0370-2693(93)90252-D)

[133] Bogoyavlenski OI, Novikov SP. 1973 Singularities of the cosmological model of the Bianchi IX type according to the qualitative theory of differential equations. Sov. Phys. JETP. **37**, 747-755.

[134] Wainwright J, Ellis GFR. 1993 Dynamical systems in cosmology. Cambridge, UK: CUP.

[135] Ringstrom H. 2000 Curvature blow up in Bianchi VIII and IX vacuum spacetimes. Class. Quant. Grav. **17**, 713-731. (10.1088/0264-9381/17/4/301)

[136] Barrow JD. 1978 Quiescent cosmology. Nature **272**, 211-215. (10.1038/272211a0)

[137] Collins CB, Hawking SW. 1973 Why is the universe isotropic? Astrophys. J. **180**, 317-334. (10.1086/151965)

[138] Goode SW, Wainwright J. 1985 Isotropic singularities in cosmological models. Class. Quant. Grav. **2**, 99-115. (10.1088/0264-9381/2/1/010)

[139] Hohn PA, Scott SM. 2009 Encoding cosmological futures with conformal structures. Class. Quant. Grav. **26**, 035019. (10.1088/0264-9381/26/3/035019)

[140] Christodoulou D. 1999 On the global initial value problem and the issue of singularities. Class. Quant. Grav. Class. Quantum Grav. **16**, A23. (10.1088/0264-9381/16/12A/302)

[141] Penrose R. 2004 The Road to Reality. London, UK: Bodley Head.

[142] Witten E. 1998 Anti-de Sitter space and holography. Adv. Theor. Math. Phys. **2**, 253-291. (10.4310/ATMP.1998.v2.n2.a2)

[143] Barrow JD, Tipler FJ. 1988 Action principles in nature. Nature **331**, 31-34. (10.1038/331031a0)

[144] Barrow JD. 2020 Finite action principle revisited. Phys. Rev. D **101**, 023527. (10.1103/PhysRevD.101.023527)

[145] Lehners J-L, Stelle KS. 2019 Safe beginning for the Universe? Phys. Rev. D **100**, 083540. (10.1103/PhysRevD.100.083540)

[146] Coleman S. 1985 Aspects of symmetry, pp. 344–345. Cambridge, UK: CUP.

